# Carvedilol Confers Ferroptosis Resistance in HL-1 Cells by Upregulating GPX4, FTH1, and FTL1 and Inducing Metabolic Remodeling Under Hypoxia/Reoxygenation

**DOI:** 10.3390/antiox14010007

**Published:** 2024-12-24

**Authors:** Yi-Chin Li, Mei-Ling Cheng

**Affiliations:** 1 Graduate Institute of Biomedical Sciences, College of Medicine, Chang Gung University, Taoyuan City 33302, Taiwan; d0901101@cgu.edu.tw; 2 Metabolomics Core Laboratory, Healthy Aging Research Center, Chang Gung University, Taoyuan City 33302, Taiwan; 3 Clinical Metabolomics Core Laboratory, Chang Gung Memorial Hospital, Taoyuan City 33305, Taiwan; 4 Department of Biomedical Sciences, College of Medicine, Chang Gung University, Taoyuan City 33302, Taiwan

**Keywords:** cardiomyocytes, hypoxia/reoxygenation, Carvedilol, lipid peroxidation, ferroptosis, GPX4, FTH1, FTL1

## Abstract

Hypoxia/reoxygenation (HR) often occurs under cardiac pathological conditions, and HR-induced oxidative stress usually leads to cardiomyocyte damage. Carvedilol, a non-selective β-blocker, is used clinically to treat cardiac ischemia diseases. Moreover, Carvedilol has also been reported to have an antioxidant ability by reducing lipid peroxidation. However, the mechanism of Carvedilol to inhibit lipid peroxidation is still elusive. To explore the protective mechanism of Carvedilol to resist lipid peroxidation on cardiomyocytes, HL-1 cells were cultured under normoxia, hypoxia, and HR and treated with Carvedilol to investigate the alteration on metabolism, protein expression, and mRNA level to explain its oxidative mechanism. The study found that Carvedilol upregulated glutathione peroxidase 4 (GPX4) protein expression to resist HR-induced lipid peroxidation by metabolic remodeling under HR. Also, Carvedilol promoted ferroptosis-related genes, ferritin heavy chain 1 (*FTH1*) and ferritin light chain 1 (*FTL1*) mRNA levels, to reduce lipid peroxidation under both hypoxia and HR. In conclusion, our study explores a mechanism by which Carvedilol inhibits ferroptosis by upregulating GPX4, *FTH1*, and *FTL1* levels to downregulate lipid peroxidation under HR. The study provides a potential strategy for using Carvedilol in clinical applications, inspiring further research and development in the area of heart diseases.

## 1. Introduction

Cardiomyocytes require an ample oxygen supply to maintain their physiological functions and metabolism. Consequently, the deficiency of oxygen caused by hypoxia and hypoxia/reoxygenation (HR) results in impaired function and metabolic disturbances [1,2,3]. Hypoxia and HR also trigger excessive reactive oxygen species (ROS) production, leading to cardiac dysfunction, oxidative injury, and cell death [1,4]. Under hypoxia, ROS primarily originates from the mitochondria due to Ca^2+^ overload mediated by the Na^+^/Ca^2+^ exchanger [2]. Hypoxia stimulates hypoxia-inducible factor (HIF) expression, promoting ROS generation [5]. Moreover, excessive ROS can damage lipids, particularly unsaturated fatty acids, leading to lipid peroxidation [6,7,8]. Hypoxia upregulates the expression of hypoxia-inducible lipid droplet-associated protein (HILPDA) and interacts with ROS to accelerate lipid peroxidation [9]. On the other hand, HR is a common injury-induced cardiac cell disruption, which affects ionic homeostasis, generates ROS, and causes mitochondrial damage, ultimately leading to cell death [10]. After hypoxia, the reoxygenation also causes rapid ROS production and exacerbates lipid peroxidation [7,11]. It also leads to the downregulation of nuclear factor E2-related factor 2 (NRF2) in cardiomyocytes under HR, resulting in increased Fe^2+^ level and enhanced lipid peroxidation [12].

Ferroptosis is an oxidative stress-induced programmed cell death (PCD) characterized by iron-dependent lipid peroxidation [13]. Glutathione peroxidase 4 (GPX4) is the only GPX protein capable of inhibiting ferroptosis [14,15]. Previous studies have demonstrated that cardiomyocytes are susceptible to ferroptosis due to increased lipid peroxidation, as evidenced by the accumulation of malondialdehyde (MDA) and decreased GPX4 expression [16,17]. Also, ferroptosis can be regulated by ferritin heavy chain 1 (FTH1) and ferritin light chain 1 (FTL1), as ferroptosis inhibitory genes modulate the storage of iron ions [18]. Consequently, elucidating the mechanisms by which cardiomyocytes resist ferroptosis is crucial.

In recent years, metabolic alterations that reduce fatty acid oxidation have been a potential treatment of cardioprotective effects under hypoxia and HR, such as β-blockers [5]. β-blockers are a common heart failure treatment by blocking β-receptors to reduce cardiac output and apoptosis [19]. Different from other β-blockers, Carvedilol is a non-selective β-blocker, which can block both α- and β-receptors to induce negative inotropy and peripheral vasodilation [20]. Also, Carvedilol can inhibit the cyclic adenosine monophosphate/protein kinase A (cAMP/PKA) pathway to ameliorate heart failure, hypertension, and cardiac ischemia diseases [20]. Most notably, Carvedilol is the only β-blocker with antioxidant properties to prevent oxidative damage [21]. Pre-treatment with Carvedilol elevates glutathione (GSH) level and inhibits oxidized glutathione (GSSG) formation to counteract hydrogen peroxide (H_2_O_2_)-induced oxidative stress [22]. In the high glucose-induced oxidative stress condition, Carvedilol activates the NRF2/antioxidant response element (ARE) pathway to shield retinal pigment epithelial cells from oxidative injury [23]. Additionally, Carvedilol upregulates *miR-133* and heat shock protein 60 (HSP60) expression to resist oxidative stress in rat neonatal cardiomyocytes [24,25]. In addition, Carvedilol can shift the metabolism from fatty acid oxidation to glucose utilization, reducing lipid peroxidation damage [5]. Also, Carvedilol inhibits lipid peroxidation by decreasing the expression of MDA [23,26]. Although Carvedilol has been clinically demonstrated to significantly reduce cardiac injury, and the mechanisms underlying this protective effect have been elucidated, the detailed mechanism of how Carvedilol performs its antioxidant ability to inhibit lipid peroxidation oxidative damages and ferroptosis via gene regulation, protein expression, and metabolic modulation, remains unclear.

To investigate the stable control of Carvedilol antioxidant ability under prolonged HR, this study explores the mechanism of Carvedilol to resist lipid peroxidation and ferroptosis in HL-1 cells under long-term normoxia, hypoxia, and HR. Therefore, we analyzed the metabolism remodeling, gene regulation, and protein expression of HL-1 mouse cardiomyocytes in Carvedilol treatment under normoxia (21% O_2_ 8 h followed by 21% O_2_ 8 h with 10 µM Carvedilol treatment), hypoxia (1% O_2_ 8 h followed by 1% O_2_ 8 h with 10 µM Carvedilol treatment), and HR (1% O_2_ 8 h followed by 21% O_2_ 8 h with 10 µM Carvedilol treatment) [10,22,25,27]. And we mainly compared HR to normoxia and hypoxia. Our findings reveal that Carvedilol regulates different strategies to inhibit lipid peroxidation and ferroptosis in HL-1 cells under HR.

## 2. Materials and Methods

### 2.1. Cell Culture, Hypoxia and HR Conditions, and Carvedilol Treatment

HL-1 atrial myocytes (Research Resource Identifier (RRID): CVCL_0303) were cultured in Claycomb medium (51800C, Sigma-Aldrich, St. Louis, MO, USA) supplemented with 10% fetal bovine serum (Life Technologies, Carlsbad, CA, USA), 1 mM norepinephrine (Sigma-Aldrich), 2 mM glutamine (Life Technologies), and 1 mM penicillin/streptomycin (Life Technologies) under 5% CO_2_ in a 37 °C incubator (also served as normoxia) [28,29]. Hypoxia (Sci-tive Dual, Baker Ruskinn, Sanford, ME, USA) was set to 1% O_2_, 5% CO_2_, and 94% N_2_ at 37 °C. HR was set to 8 h hypoxia followed by 8 h reoxygenation. Carvedilol (10 µM, Sigma-Aldrich) was dissolved in 0.1% dimethyl sulfoxide (DMSO) and was given in the latter 8 h [10,22,25,27]. An HL-1 cells at 0 h served as the control.

### 2.2. Analysis for Cellular ROS, Lipid Peroxidation, and Cell Death

Cellular ROS, lipid peroxidation, and cell death were detected by using CellROX Deep Red reagent (Thermo Fisher Scientific, Waltham, MA, USA), C11 BODIPY 581/591 reagent (Thermo Fisher Scientific), and propidium iodide (PI, Sigma-Aldrich) [30]. Cellular ROS and lipid peroxidation were analyzed by LEICA STELLARIS8 (LECIA, Wetzlar, Germany), and cell death was analyzed by LionHeart FX (Agilent Technologies, Santa Clara, CA, USA). In addition, 5 µg/mL Hoechst 333432 was used in all staining analysis. The data were compared to the 0 h control.

### 2.3. Western Blot Analysis

Protein samples were extracted using RIPA lysis buffer, and protein concentration was quantified by a Bradford assay. Protein samples were detected by electrophoresis with a polyvinylidene difluoride (PVDF) membrane. The membrane was blocked with 5% non-fat milk and then incubated with primary antibodies at 4 °C overnight. Primary anti-bodies used in the study are listed as follows: rabbit secondary antibodies (BIO-DOC, BAB2103, 1:10,000) for HIF-1α (Cell Signaling Technology, Danvers, MA, USA, 14,179, 1:1000), Bcl-2 (Cell Signaling Technology, 2870, 1:1000), Bax (abcam, Cambridge, UK, ab32503, 1:4000), and Actin (Sigma-Aldrich, A2103, 1:10,000, served as loading control) and goat secondary anti-bodies (Santa Cruz Biotechnology, Dallas, TX, USA, sc-2020, 1:10,000) for GPX4 (abcam, ab116703, 1:1000). Proteins were explored by ECL chemiluminescence and quantified of band intensity by using ImageJ 1.54d. The relative protein expression was normalized to Actin and compared to the 0 h control.

### 2.4. Analysis for Cell Viability

For the cell viability analysis, after incubating under normoxia, hypoxia, and HR for 16 h, CCK-8 solution (Polycreatives, Kaohsiung City, Taiwan) was added to cells. After 2 h incubation, cells were taken to read the O.D at 450 nm by an ELISA reader, and the relative cell reduction was calculated by comparing the results in hypoxia or HR to normoxia with Carvedilol treatment or not, respectively.

### 2.5. Analysis of Extracellular Metabolites by Nuclear Magnetic Resonance (NMR)

Cell medium samples were mixed with D_2_O solution containing 1 mM TSP and 3 mM NaN_3_ (1:1) and then loaded into the NMR tubes. Analysis of prepared samples was performed by a Bruker Avance III HD 600 MHz NMR system (Bruker, Billerica, MA, USA). The NMR data were processed and integrated by the software TopSpin 3.6.1 (Bruker), then compared to the 0 h control.

### 2.6. Analysis for Fatty Acid Oxidation (FAO) Oxygen Consumption Rate (OCR) by Seahorse XF24 Analyzer

For the mitochondrial OCR of fatty acid oxidation analysis, after incubating under normoxia, hypoxia, and HR, cells were incubated in FAO assay buffer for 1 h. Before subjecting to Seahorse XF24 analyzer, 1 mM palmitate was added in 1% BSA to cells, then the OCR was analzyed followed by 4 µM etomoxir (Cayman Chemical, Ann Arbor, MI, USA), 1 µM oligomycin (Cayman Chemical), 1 µM FCCP (Cayman Chemical), 1 µM rotenone (Sigma-Aldrich), and 1 µM antimycin A (Sigma-Aldrich). The OCR was normalized by crystal violet staining absorbance at 570 nm.

### 2.7. Analysis of Targeted Intracellular Metabolites by Liquid Chromatography Coupled with Tandem Mass Spectrometry (LC-MS/MS)

For targeted intracellular metabolite analysis, the cell extraction and LC-MS/MS were carried out as previously described [31], but the samples were instead dissolved in 100% ddH_2_O in the analysis. In NADP and NADPH analysis, the LC-MS/MS analysis condition was also based on that previously described [31], but the analysis method was modified by the following: cell samples were extracted with 10 mM KOH in 80% (*v*/*v*) MeOH/H_2_O and were dissolved in 50% (*v*/*v*) ACN/H_2_O. The analyzed column was Atlantis BEH Z-HILIC (100 × 2.1 mm, particle size of 1.7 µm, Waters Corp., Milford, MA, USA) and was set to 30 °C. The mobile phase consisted of solvent A (15 mM ammonium bicarbonate) and solvent B (15 mM ammonium bicarbonate/90% ACN/H_2_O). The flow rate was set to 0.5 mL/min, and the linear gradient of solvents was set as follows: linear gradient 90–65% B, 5 min; 65% B, 1 min; 65–90% B, 0.5 min; and 90% B for an additional 3 min. The ionization mode was ESI in positive ion mode. All metabolites were analyzed by using MassLynx software 4.2 (Waters Corp.) and were compared to the 0 h control.

### 2.8. Analysis of Untargeted Intracellular Metabolites by Ultrahigh-Performance Liquid Chromatography Time-of-Flight Mass Spectrometry (UPLC-TOF-MS)

For untargeted intracellular metabolite analysis, the cell extraction and UPLC-TOF-MS were carried out as previously described [32], but the mass system was modified to Vion IMS QTof. All untargeted data were processed and analyzed by using Progenesis QI 2.4 (Waters Corp.), MetaboAnalyst 6.0, and the Human Metabolome Database (HMDB) for a comprehensive understanding of the cellular metabolites, compared to the 0 h control.

### 2.9. Quantitative Polymerase Chain Reaction (qPCR)

Cell samples were lysed by using TRIzol solution (Life Technologies), and the total RNA was extracted by chloroform, isopropanol, and ethanol, and then quantified using NanoDrop (Implen, Munich, Germany). The cDNA was synthesized from the extracted RNA using a RevertAid first-strand cDNA synthesis kit (Thermo Fisher Scientific) following the manufacturer’s instructions. The cDNA sample was mixed with EvaGreen reagent (BIO-RAD, Hercules, CA, USA) and accessed to the qPCR analysis. The relative mRNA levels were calculated with the 2^−ΔΔCT^ calculations with beta-Actin as the endogenous reference [31]. Specific primers were used to amplify the cDNA (Table 1).

### 2.10. Statistical Analysis

In each experiment, we conducted three independent replicates, with each replicate completed in triplicate. Data were presented as the mean ± SD in three independent experiments. Statistical comparisons were made by an analysis of variance (ANOVA) with Student–Newman–Keuls (SNK) test with IBM SPSS 20.0 software (IBM, Armonk, NY, USA). The asterisks in the figures indicate statistical significance (* *p* ≤ 0.05; ** *p* ≤ 0.01; *** *p* ≤ 0.001).

## 3. Results

### 3.1. Hypoxia and HR Induced Oxidative Stress and Lipid Peroxidation

**Figure 1:**
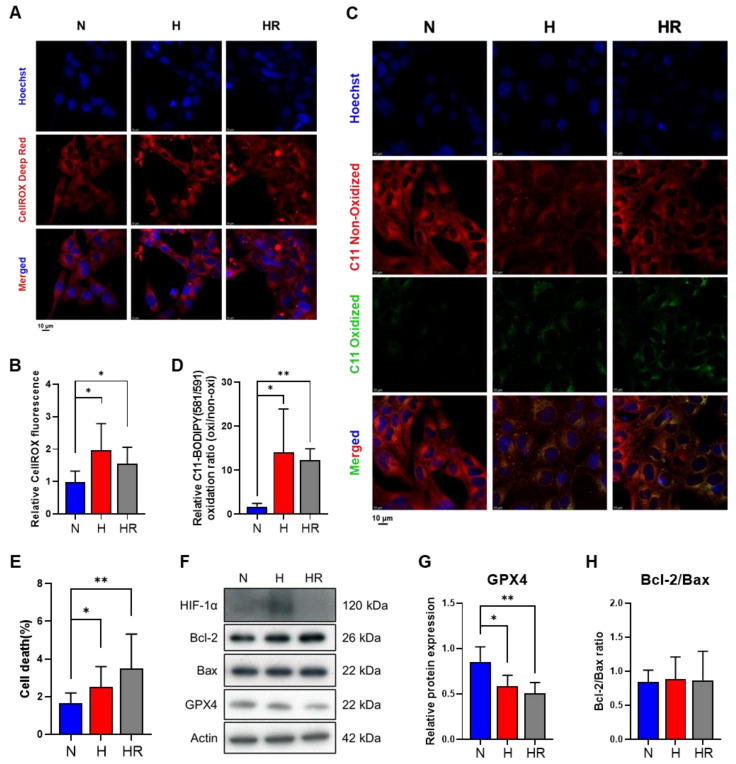
Effects of normoxia, hypoxia, and HR on HL-1 cells. HL-1 cells were cultured under normoxia, hypoxia, and HR, respectively. Cellular ROS levels were analyzed by 2.5 µM CellROX Deep Red (**A**) and the fluorescence intensity was detected. (**B**) Lipid peroxidation levels were measured by 1 µM C11 BODIPY 581/591 (**C**) and the fluorescence intensity ratios of C11 oxidized (green) and C11 non-oxidized (red) BODIPY were analyzed (**D**). Scale bar: 10 μm. The cell death percentage of HL-1 cells was stained by 1 µg/mL PI and analyzed by LionHeart FX (**E**). The protein expression of HIF-1α, Bcl-2, Bax, GPX4, and the Bcl-2/Bax ratio was measured by using Western blot analysis (**F**–**H**). Data of HL-1 cells at 0 h were 1. Data were presented as mean ± SD in three independent experiments, with each group completed in triplicate. Statistical analysis by ANOVA with SNK test: * *p* ≤ 0.05; ** *p* ≤ 0.01. N: normoxia; H: hypoxia; HR: hypoxia/reoxygenation; HIF-1α: hypoxia-inducible factor-1α; GPX4: glutathione peroxidase 4.

To confirm the effects of hypoxia and HR on HL-1 cells, cellular ROS and lipid peroxidation were first identified. The CellROX staining revealed that hypoxia and HR significantly increased cellular ROS levels (Figure 1A,B). Moreover, the lipid peroxidation levels, by C11 BODIPY 581/591 staining, were higher significantly under HR (Figure 1C,D). On the other hand, both hypoxia and HR increased the percentage of cell death (Figure 1E). The result suggested that both hypoxia and HR induced oxidative stress and caused cell death compared to normoxia, and the results suggested that HR contributed to higher lipid peroxidation levels in HL-1 cells.

To further confirm the results that HR induced lipid peroxidation, we detected GPX4 protein expression (Figure 1F). Compared to normoxia, the reduced protein expression of GPX4 was correlated to the increased level of lipid peroxidation under hypoxia and HR (Figure 1F,G). The protein expression ratio of Bcl-2 and Bax showed no differences among three conditions, suggesting that Bcl-2 and Bax were not the main regulators for cell death under hypoxia and HR (Figure 1F,H). On the other hand, HIF-1α protein expression was only observed under hypoxia, indicating the hypoxia system worked in HL-1 cells (Figure 1F). Based on the results, it supported that HR led to the reduced expression of GPX4 to induce lipid peroxidation in HL-1 cells.

### 3.2. Carvedilol Promoted Lipid Peroxidation Reduction Under HR

**Figure 2:**
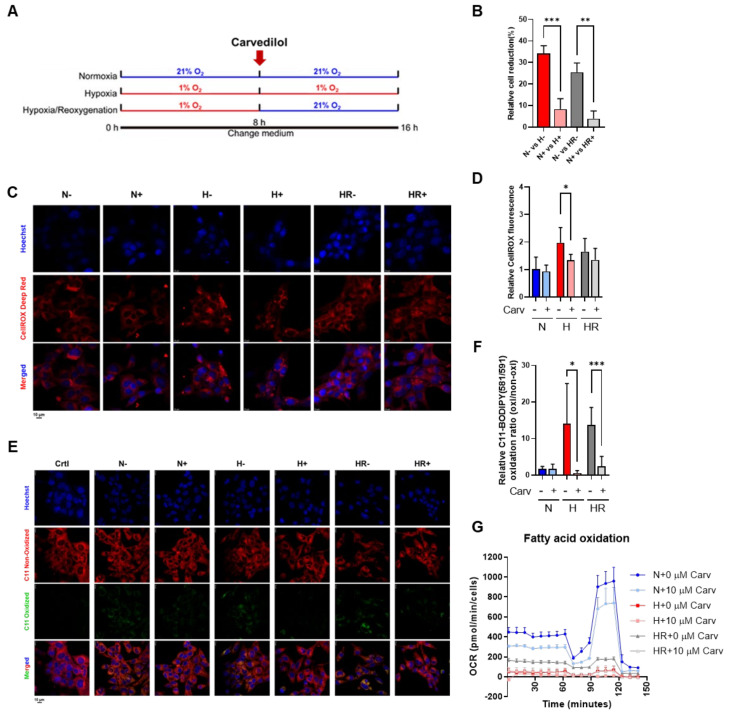
Effects of Carvedilol on cellular oxidative stress and lipid peroxidation under normoxia, hypoxia, and HR. (**A**) Experimental schematic diagram of Carvedilol treatment on HL-1 cells under normoxia, hypoxia, and HR. A total of 10 µM Carvedilol was used in the latter 8 h. After the Carvedilol treatment, HL-1 cells were subjected to (**B**) cell reduction analysis, (**C**,**D**) CellROX Deep Red fluorescence intensity detection, and (**E**,**F**) C11 BODIPY 581/591 fluorescence intensity analysis. Scale bar: 10 μm. (**G**) The Seahorse XF24 analyzer was used to assess the OCR of fatty acid oxidation, and the OCR was normalized with a relative cell number fold change. Data of HL-1 cells at 0 h were 1. Data were presented as mean ± SD in three independent experiments, with each group completed in triplicate. Statistical analysis by ANOVA with SNK test: * *p* ≤ 0.05; ** *p* ≤ 0.01; *** *p* ≤ 0.001. Carv: Carvedilol; N: normoxia; H: hypoxia; HR: hypoxia/reoxygenation.

To test the influence of oxidative stress induced by hypoxia and HR in HL-1 cells, Carvedilol was administered for the last 8 h among three conditions (Figure 2A). We analyzed the relative cell reduction by using a CCK-8 assay and indicated that Carvedilol significantly decreased the cell reduction percentage under hypoxia and HR, supporting that Carvedilol actually prevents cell reduction under hypoxia and HR (Figure 2B).

In oxidative stress monitoring, Carvedilol treatment under hypoxia significantly reduced (~32.2%) cellular ROS and decreased (~96.0%) lipid peroxidation (Figure 2C–F). On the other hand, Carvedilol treatment under HR significantly reduced lipid (~82.6%) peroxidation, while cellular ROS showed slightly decreased levels (~18.2%) (Figure 2C–F). It was interesting that Carvedilol actually decreased cellular ROS compared to HR, while Carvedilol significantly decreased lipid peroxidation under both hypoxia and HR. The results supported that Carvedilol treatment to HL-1 cells can significantly inhibit lipid peroxidation to prevent the cells from oxidative stress and damage, especially under HR. On the other hand, Carvedilol inhibited the fatty acid oxidation OCR under three conditions (Figure 2G), suggesting that Carvedilol reduced lipid utilization to diminish mitochondrial oxygen consumption and ROS formation.

### 3.3. Carvedilol Activated GPX4 Expression by Upregulating Cysteine and Methionine Metabolism and GSH Metabolism to Counteract Oxidative Stress Under HR

**Figure 3:**
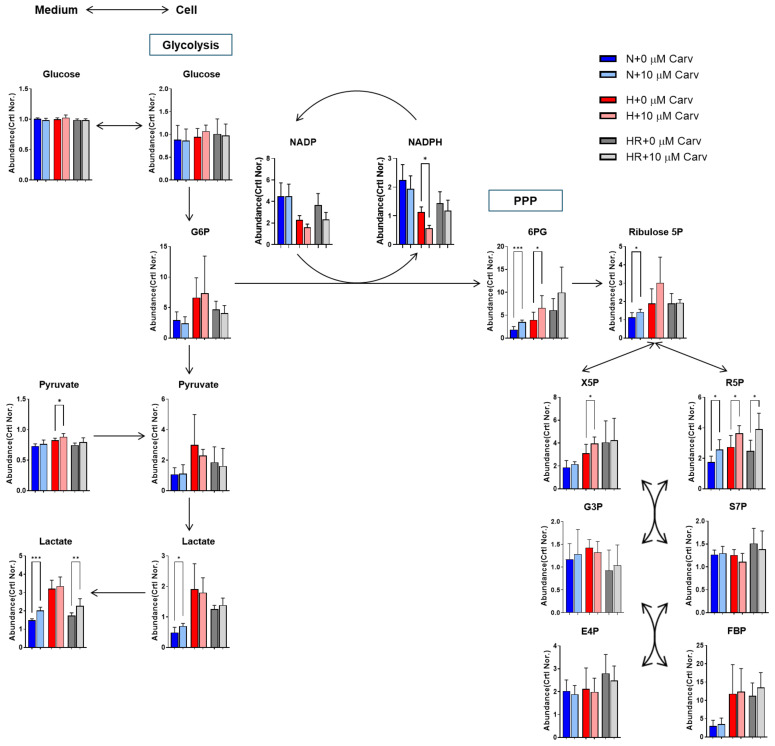
Effects of Carvedilol on glycolysis and PPP under normoxia, hypoxia, and HR. After the Carvedilol treatment, HL-1 cells were subjected to metabolite analysis for glycolysis and PPP. In glycolysis, intracellular and extracellular abundance of glucose, pyruvate, and lactate were measured by LC-MS/MS and NMR. In PPP, PPP metabolites, NADP, and NADPH were assessed for LC-MS/MS analysis. All data above were normalized with 0 h control. Data were presented as mean ± SD in three independent experiments, with each group completed in triplicate. Statistical analysis by ANOVA with SNK test: * *p* ≤ 0.05; ** *p* ≤ 0.01; *** *p* ≤ 0.001. Crtl: control; Carv: Carvedilol; N: normoxia; H: hypoxia; HR: hypoxia/reoxygenation; PPP: pentose phosphate pathway; G6P: glucose 6-phosphate; 6PG: 6-phosphogluconate; Ribulose 5P: Ribulose 5-phosphate; R5P: ribose 5-phosphate; X5P: xylulose 5-phosphate; S7P: sedoheptulose 7-phosphatase; G3P: glyceraldehyde 3-phosphate; FBP: fructose 1,6-bisphosphate; E4P: erythrose 4-phosphate.

To investigate the antioxidant mechanism of Carvedilol, we first focused on metabolic alternation. As hypoxia has been reported to enhance the flux of glycolysis [2], we detected the abundance of primary metabolites in glycolysis, such as glucose, pyruvate, and lactate. In Figure 3 for glycolysis, glucose and pyruvate abundances showed no difference in Carvedilol treatment under three conditions. Carvedilol treatment increased both intracellular and extracellular abundances of lactate under normoxia and HR but showed no differences under hypoxia, suggesting that Carvedilol might enhance the flux of glycolysis under normoxia and HR.

Next, while the pentose phosphate pathway (PPP) has the oxidative branch to generate NADPH to resist oxidative stress [33], we then analyzed the PPP metabolite abundances. In Figure 3 for PPP, Carvedilol treatment elevated the abundance of 6-phosphogluconate (6PG), xylulose 5-phosphate (X5P), and ribose 5-phosphate (R5P) under three conditions. However, NADP and NADPH abundances showed no difference in Carvedilol treatment under normoxia and HR but were decreased under hypoxia. These results indicated that Carvedilol enhanced most of PPP upstream metabolite abundances under HR but did not affect NADP and NADPH abundance, revealing that Carvedilol performed its antioxidant ability by other mechanisms.

**Figure 4:**
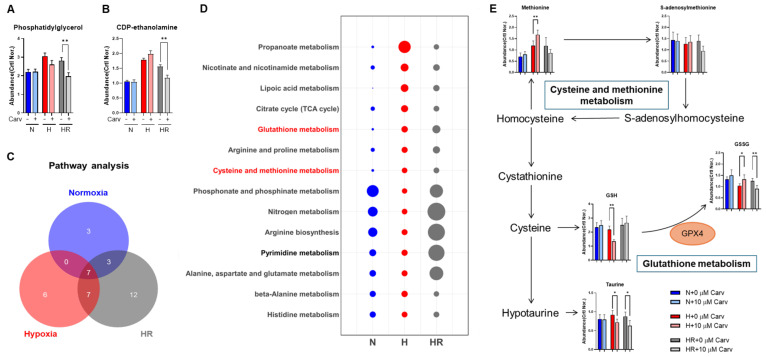
Effects of Carvedilol on untargeted metabolic analysis under normoxia, hypoxia, and HR. The untargeted UPLC-TOF-MS analysis assessed (**A**,**B**) the abundance of phosphatidylglycerol and CDP-ethanolamine. (**C**) Venn diagram of pathway analysis based on the untargeted metabolites analyzed results showed the intersection with or without Carvedilol under normoxia(blue), hypoxia (red), and HR (gray), and (**D**) bubble chart showing 14 significant pathways under hypoxia and hypoxia/reoxygenation compared to normoxia. The dot size was proportional to the correlation with metabolism, and the dot color represented the intersection with or without Carvedilol under normoxia (blue), hypoxia (red), and HR (gray). (**E**) Cysteine and methionine metabolism and glutathione metabolism was predicted to be the potential metabolic pathway regulated by Carvedilol under normoxia, hypoxia, and HR. All data above were normalized with 0 h control. Data were presented as mean ± SD in three independent experiments, with each group completed in triplicate. Statistical analysis by ANOVA with SNK test: * *p* ≤ 0.05; ** *p* ≤ 0.01. Crtl: control; Carv: Carvedilol; N: normoxia; H: hypoxia; HR: hypoxia/reoxygenation; GSH: glutathione; GSSG: oxidized glutathione; GPX4: glutathione peroxidase 4.

To elucidate the potential metabolic mechanisms, we performed an untargeted metabolic analysis. First, we found that the abundance of phosphatidylglycerol and CDP-ethanolamine, two lipid metabolites related to lipid synthesis [34,35], were only reduced in Carvedilol treatment under HR (Figure 4A,B). Next, we accessed the untargeted metabolites to pathway enrichment analysis and analyzed the pathway correlation between no Carvedilol treatment and Carvedilol treatment under three conditions, respectively. According to the untargeted metabolic pathway analysis, 38 significant metabolic pathways were identified (Figure 4C), and we picked up 14 metabolic pathways which were significant under hypoxia and HR (Figure 4D). In the 14 metabolic pathways, we found that cysteine and methionine metabolism and GSH metabolism showed a linkage to GPX4 expression (Figure 4E). Therefore, we predicted that Carvedilol performed its antioxidant ability through promoting cysteine and methionine metabolism and GSH metabolism to regulate GPX4 expression.

**Figure 5:**
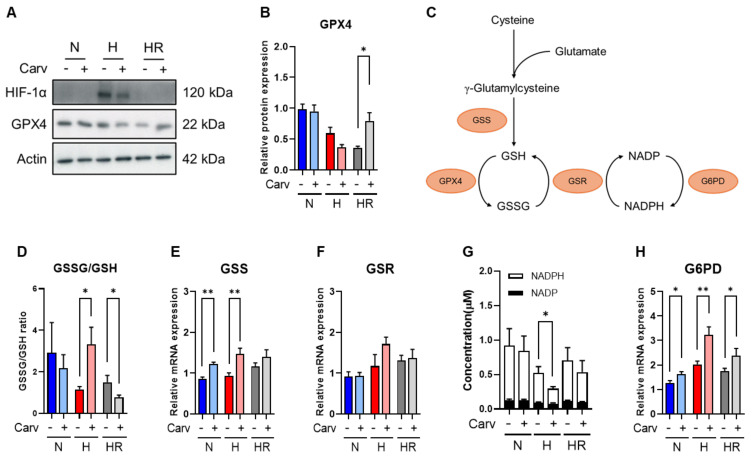
Effects of Carvedilol on GPX4 regulation. (**A**,**B**) Protein expression of HIF-1α and GPX4 in HL-1 cells in Carvedilol treatment was measured by Western blot analysis. (**C**) Metabolism from cysteine to GSH and GSSG. (**D**) The ratio of GSSG and GSH; (**E**,**F**) the *GSS* and *GSR* mRNA levels by qPCR; (**G**) the total concentration of NADP and NADPH; (**H**) the *G6PD* mRNA level. Data were presented as mean ± SD in three independent experiments, with each group completed in triplicate. Statistical analysis by ANOVA with SNK test: * *p* ≤ 0.05; ** *p* ≤ 0.01. N: normoxia; H: hypoxia; HR: hypoxia/reoxygenation, Carv: Carvedilol; HIF-1α: hypoxia-inducible factor-1α; GPX4: glutathione peroxidase 4; GSH: glutathione; GSSG: oxidized glutathione; *GSS*: glutathione synthetase; *GSR*: glutathione disulfide reductase; NADP: nicotinamide adenine dinucleotide phosphate; *G6PD*: glucose-6-phosphate dehydrogenase.

To confirm the prediction, we validate the GPX4 expression. Carvedilol treatment under HR significantly increased (~1.21%) GPX4 protein expression, and the GPX4 expression activated by Carvedilol under HR was highly comparable to that under normoxia (Figure 5A,B). In contrast, unlike the expression under HR, GPX4 expression under hypoxia showed no difference in Carvedilol treatment, and the result might contribute to the lower expression of HIF-1α protein under hypoxia in Carvedilol treatment (Figure 5A,B) [36]. Based on the results, Carvedilol was revealed to enhance GPX4 protein expression under HR. To further validate the upregulation of GPX4, we analyzed the metabolite abundance and mRNA levels of GSH metabolism (Figure 5C). First, Carvedilol treatment under HR decreased GSSG abundance but did not affect GSH abundance, and Carvedilol treatment under hypoxia showed GSH elevation and GSSG reduction (Figure 4E). The GSSG/GSH ratio in Carvedilol treatment also showed an increase under hypoxia, a decreased ratio under HR, and no difference under normoxia (Figure 5D). On the other hand, Carvedilol markedly enhanced the level of mRNA levels of GSH synthetase (*GSS*) under hypoxia, with a slight activation in the *GSS* level under HR (Figure 5E). In contrast, the GSH-disulfide reductase (*GSR*) mRNA level showed no difference among the three conditions (Figure 5F). The increased level of *GSS* supported the upregulation of cysteine and methionine metabolism in Carvedilol treatment.

Furthermore, while the conversion of GSSG to GSH was accompanied with an NADPH to NADP conversion, we also analyzed the concentration of NADP and NADPH and found that the total concentration of NADP and NADPH under normoxia and HR showed no changes in Carvedilol treatment but was reduced under hypoxia (Figure 5G). Carvedilol also activated glucose-6-phosphate dehydrogenase (*G6PD*) mRNA levels among three conditions (Figure 5H). These results correlated to the elevated PPP in Carvedilol (Figure 3). As a result, we suggested that GSH and GSSG abundances under HR was contributed to by the unchanged abundance of NADPH, and we verified that activated GPX4 protein expression in Carvedilol treatment under HR was accompanied with GSH metabolism to maintain the GSH abundance.

### 3.4. Carvedilol Activated FTH1 and FTL1 to Resist Lipid Peroxidation

**Figure 6:**
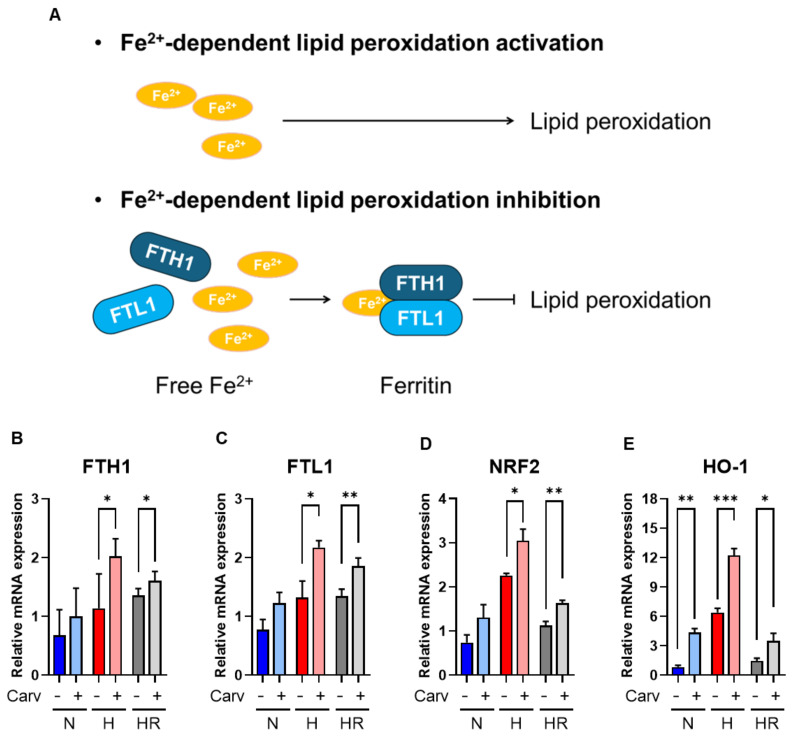
Effects of Carvedilol on Fe^2+^-dependent lipid peroxidation regulation. (**A**) The regulation of Fe^2+^ to lipid peroxidation. (**B**,**C**) mRNA levels of *FTH1* and *FTL1* involved in the Fe^2+^-dependent lipid peroxidation regulation. (**D**,**E**) *NRF2* and *HO-1* mRNA levels. Data were presented as mean ± SD in three independent experiments, with each group completed in triplicate. Statistical analysis by ANOVA with SNK test: * *p* ≤ 0.05; ** *p* ≤ 0.01; *** *p* ≤ 0.001. N: normoxia; H: hypoxia; HR: hypoxia/reoxygenation, Carv: Carvedilol; *FTH1*: ferritin heavy chain 1; *FTL1*: ferritin light chain 1; *NRF2*: nuclear factor E2-related factor 2; *HO-1*: heme oxygenase-1.

To further confirm that the Carvedilol antioxidant ability can resist Fe^2+^-dependent lipid peroxidation and ferroptosis, we analyzed the *FTH1* and *FTL1* mRNA levels. The accumulation of Fe^2+^ induced the activation of Fe^2+^-dependent lipid peroxidation, and the expression of FTH1 and FTL1 can combine to Fe^2+^ to form ferritin, resulting in the decrease of Fe^2+^ to inhibit lipid peroxidation and ferroptosis (Figure 6A) [37,38]. In this study, the *FTH1* mRNA level was increased significantly in Carvedilol treatment under hypoxia (~48.7%) and HR (~14.7%). Also, the *FTL1* mRNA level was enhanced significantly in Carvedilol treatment under hypoxia (~57.9%) and HR (~37.0%) (Figure 6B,C). These results supported that Carvedilol activated *FTH1* and *FTL1* mRNA to resist Fe^2+^-dependent lipid peroxidation and ferroptosis.

Lipid peroxidation has been reported to be suppressed by increased levels of NRF2 and its downstream target genes, *GSR*, *GSS*, *GPX4*, *FTH1*, and *FTL1* to perform ferroptosis resistance [18,39]. As a result, we hypothesized that the induction of *GPX4*, *GSS*, *GSR*, *FTH1*, and *FTL1* was associated with *NRF2* activation. In our study, the *NRF2* mRNA level was significantly increased among three conditions (Figure 6D), and most *NRF2*-targeted genes under hypoxia and HR were activated in our study (Figure 5E and Figure 6B,C). On the other hand, we also confirmed that the *HO-1* mRNA level, another *NRF2*-conducted gene, was significantly enhanced in Carvedilol treatment under three conditions (Figure 6E). Intriguingly, while Carvedilol treatment decreased GPX4 expression under hypoxia, it did not align with the increased *NRF2* levels. Conversely, under HR conditions, both exhibited a consistent pattern, supporting the notion that Carvedilol might regulate GPX4 via additional *NRF2*-independent pathways. These results supported that Carvedilol upregulated *NRF2* to activate *FTH1*, and *FTL1* to inhibit lipid peroxidation and ferroptosis.

## 4. Discussion

**Figure 7:**
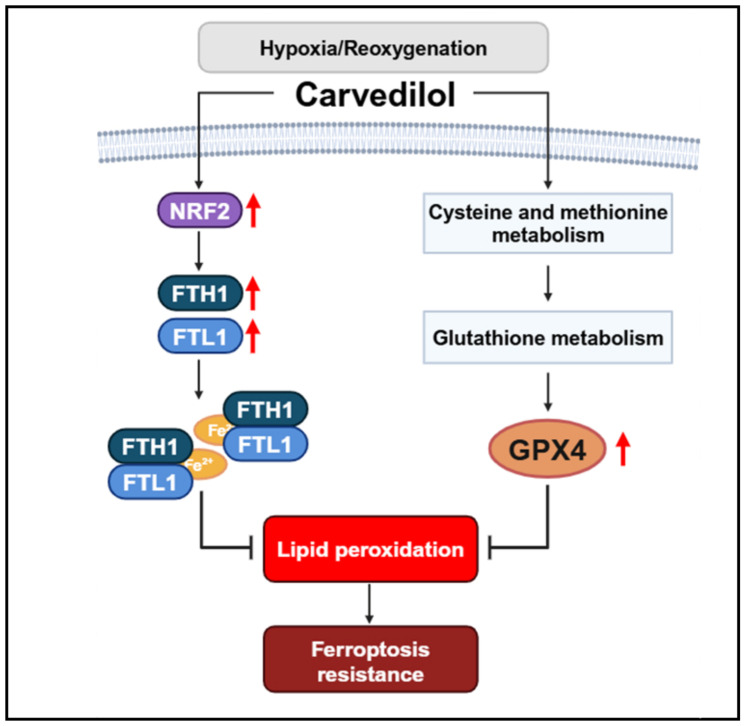
Carvedilol promotes ferroptosis resistance through upregulation of GPX4, *FTH1*, and *FTL1* to downregulate lipid peroxidation and ferroptosis under HR.

By using metabolism, genome, and protein analysis, we demonstrate that Carvedilol significantly resists lipid peroxidation and ferroptosis in HL-1 cells under HR. Our mechanistic studies proposed that Carvedilol inhibits HR-induced lipid peroxidation and ferroptosis via two different strategies, namely that (1) Carvedilol activates *NRF2* to enhance the mRNA levels of *FTH1* and *FTL1* and (2) Carvedilol activates GPX4 protein expression through upregulating cysteine and methionine metabolism and GSH metabolism (Figure 7).

Hypoxia and HR lead to cell death by apoptosis, autophagy, necroptosis, pyroptosis, and ferroptosis [40]. Apoptosis leads to Bcl-2 inhibition and Bax activation under hypoxia and HR [41,42]. However, the Bcl-2/Bax ratio in our study shows no difference compared to normoxia, suggesting that our hypoxia and HR time course may not influence apoptosis. On the other hand, hypoxia and HR actually induced cellular ROS and lipid peroxidation, but Carvedilol treatment under HR only reduced the lipid peroxidation level but not the cellular ROS level. These results made us focus on the Carvedilol antioxidant effects under HR on lipid peroxidation.

Previous studies revealed that hypoxia plays a dual role in lipid peroxidation regulation. Hypoxia induces resistance to RSL3- or erastin-induced lipid peroxidation through HIF-1α activation [30]. Also, hypoxia upregulates sentrin-specific protease 1 (SENP1), HIF-1α, and acyl-CoA synthetase long-chain family member 4 (ACSL4) to protect H9c2 cardiomyocytes from erastin-induced lipid peroxidation [43]. In contrast, hypoxia promotes the MDA level and reduces GPX4 protein expression in chronic intermittent hypoxia-induced lung injury and trophoblast cells [44,45]. Additionally, GPX4 and xCT (a glutamate/cystine antiporter) protein expression was reduced under hypoxia in AC16 cardiomyocytes, leading to lipid peroxidation upregulation [46]. According to previous studies, it can be concluded that hypoxia resists the specific drug-induced lipid peroxidation but, conversely, hypoxia alone promotes lipid peroxidation. In our study, we cultured HL-1 cells under hypoxia without treating any lipid peroxidation activators, and the results showed that hypoxia enhances the C11 BODIPY 581/591 staining level, increases phosphatidylglycerol and CDP-ethanolamine abundance, and reduces GPX4 protein expression (Figure 1 and Figure 4A,B). These results supported the fact that the hypoxia time course actually induced lipid peroxidation in HL-1 cells.

On the other hand, HR often occurs under pathological conditions and usually induces cell damage, including oxidative stress and lipid peroxidation [10]. Most studies on HR-induced oxidative stress and lipid peroxidation were especially focused on cardiomyocytes [47,48,49]. HR has been reported to promote the accumulation of Fe^2+^ levels to enhance lipid peroxidation [12]. However, while most studies indicate that both hypoxia and HR increase lipid peroxidation, it remains unclear whether reoxygenation exacerbates lipid peroxidation due to similar MDA or GPX4 levels under hypoxia and HR [48,49,50]. Our results reveal that lipid peroxidation levels and GPX4 protein expression under HR are both similar to that which is induced under hypoxia (Figure 1), suggesting that 8 h reoxygenation and the latter 8 h hypoxia promotes a certain degree of lipid peroxidation damage.

Carvedilol, a non-selective β-blocker, is a common clinical therapy to prevent the damages of heart failure, hypertension, and ischemic heart diseases by inducing negative inotropy and peripheral vasodilation [20]. Also, Carvedilol blocks β-receptor to inhibit the cAMP/PKA pathway to perform negative cardiac contractility and peripheral vasodilation to release cardiac damages [20,21]. Many studies have reported that Carvedilol has the ability to suppress ROS and lipid peroxidation. Carvedilol promotes superoxide dismutase (SOD) and NOX expression to resist ROS damage [51,52]. Pretreatment of Carvedilol resists H_2_O_2_ stress by increased GSH and decreased GSSG [22]. On the other hand, Carvedilol was considered to suppress lipid peroxidation because of a decreased MDA level [21,53]. Carvedilol can also switch the metabolism from fatty acid oxidation to glycolysis, and it may be a potential mechanism to reduce lipid peroxidation by decreasing the utilization of fatty acids [5]. In our results, Carvedilol significantly reduced lipid peroxidation levels under HR (Figure 2D–F), and the utilization of fatty acids was reduced in Carvedilol treatment among three conditions (Figure 2G). The glycolysis product, lactate, was also higher in Carvedilol treatment under HR and normoxia (Figure 3). However, lactate abundance had no difference in Carvedilol treatment under hypoxia. This result may be caused by the higher utilization of glycolysis under hypoxia. Although Carvedilol has been clinically demonstrated to significantly reduce cardiac damages, the mechanisms by which it resists oxidative stress remain unknown. Hence, a thorough understanding of Carvedilol antioxidant mechanisms will facilitate its new avenues for further investigation and potential applications.

Ferroptosis regulation can be briefly divided into two different mechanisms, namely GPX4 regulation and iron-dependent lipid peroxidation modulation [54]. GPX4 is the GPX protein that can inhibit lipid peroxidation by reducing phospholipid hydroperoxides [55,56]. GPX4 also converts GSH to GSSG, and this interaction was regulated by cysteine, the rate-limiting precursor for GPX4. Many studies have revealed that hypoxia and HR reduce GPX4 expression to enhance lipid peroxidation [9]. However, the relationship between Carvedilol and GPX4 is rarely explored. In our results, metabolic analysis revealed that Carvedilol treatment under HR upregulated cysteine and methionine metabolism (Figure 4E) and activated GPX4 expression (Figure 5E,F), supporting that Carvedilol resisted ferroptosis through GPX4 activation under HR. However, GPX4 protein expression under hypoxia did not activate in Carvedilol treatment. The reduced expression of GPX4 in Carvedilol treatment under hypoxia might be related to the decreased expression of HIF-1α. HIF-1α is an oxygen-regulated protein, which is quickly degraded in sufficient oxygen conditions and expressed in the insufficiency of oxygen [57]. Therefore, unlike HR, Carvedilol treatment under hypoxia had to consider the effects of HIF-1α. Previous studies have reported that Carvedilol can reduce HIF-1α expression [58]. However, HIF-1α has been regarded to have a dual role in lipid peroxidation regulation; HIF-1α inhibited lipid peroxidation by SENP1 activation and promoted lipid peroxidation by reducing transport subunit solute carrier family 7member 11 (SLC7A11) and GPX4 expression [9]. Based on previous studies, it is important to verify the role of HIF-1α in lipid peroxidation regulation under hypoxia. In our study, Carvedilol treatment showed a lower HIF-1α expression compared to no treatment under hypoxia, while the GPX4 expression under hypoxia was also reduced. But the lipid peroxidation level was still decreased in Carvedilol treatment under hypoxia, supporting that HL-1 cells under hypoxia might perform other mechanisms to resist lipid peroxidation.

GPX4 expression is related to GSH metabolism accompanied with GSS and GSR [36]. GPX4 acts as an enzyme to oxidize GSH into GSSG. GSS catalyzes the conversion of cysteine to GSH, and GSR catalyzes the conversion of GSSG to GSH. Therefore, GSS and GSR can contribute to the maintenance of GSH levels. In our study, Carvedilol treatment under HR stimulated the *GSS* mRNA level without altering the *GSR* mRNA level (Figure 5E,F). These results indicated that Carvedilol under HR upregulated cysteine and methionine metabolism to serve as a compensatory mechanism to maintain adequate GSH levels and sustain GPX4 activation. Furthermore, GSR promotes the conversion of NADPH to NADP. G6PD promotes the conversion of NADP to NADPH and also catalyzed the conversion of glucose 6-phosphate (G6P) to 6PG in PPP [33,36]. Our study verified the upregulation of PPP and *G6PD* mRNA levels in Carvedilol under three conditions (Figure 3 and Figure 5H), and the total concentration of NADP and NADPH showed no difference under HR. These results validated that Carvedilol activated PPP to maintain NADPH and NADP pools, thereby contributing to the stabilization of cellular GSH levels for GPX4 activation.

On the other hand, iron-dependent lipid peroxidation modulation is another important mechanism to regulate ferroptosis [54]. The accumulation of Fe^2+^ induced lipid peroxidation, while FTH1 and FTL1 can combine to Fe^2+^, forming ferritin to reduce Fe^2+^ accumulation and inhibit iron-dependent lipid peroxidation (Figure 6A). Previous studies have reported that increased FTH1 and FTL1 expression inhibited ferroptosis in tumor cells [9,30]. In our study, Carvedilol induced both *FTH1* and *FTL1* mRNA levels under hypoxia and HR. Based on the results, Carvedilol can significantly elevate *FTH1* and *FTL1* levels to inhibit Fe^2+^-dependent lipid peroxidation, which further supported Carvedilol ferroptosis resistance.

NRF2 was regarded as an effective factor against oxidative stress [23]. In previous studies, the stable expression of NRF2 promotes downstream gene transcription, further activating the following target gene expression and related pathways: (1) GSR and GSS genes expression in GSH generation, (2) GPX4 and HO-1 gene expression in ROS scavenging, and (3) *FTH1* and *FTL1* gene expression in iron metabolism [18]. On the other hand, Carvedilol activates NRF2 expression to inhibit oxidative stress and MDA levels [23]. In our study, Carvedilol activated *NRF2*-targeted genes, *GSS*, *GPX4*, *FTH1*, and *FTL1* to inhibit lipid peroxidation and ferroptosis under HR; we hypothesized that NRF2 might be a key role for Carvedilol to resist lipid peroxidation and ferroptosis. However, Carvedilol treatment under normoxia and hypoxia did not activate GPX4 expression. Under normoxia, the GPX4 expression was similar in both Carvedilol treatment and no treatment groups, supporting that GPX4 expression was actually activated under normoxia, regardless of Carvedilol treatment or not. And under hypoxia, besides the decreased HIF-1α expression in Carvedilol treatment, the decreased GPX4 expression in Carvedilol treatment might also be related to the increased GSSG/GSH ratio, suggesting that there was not enough GSH to support GPX4 expression. As a result, the elevated *NRF2* mRNA levels in Carvedilol treatment among three conditions actually served as the culminating linkage to upregulate *FTH1* and *FTL1* mRNA levels to perform lipid peroxidation and ferroptosis resistance mechanistic studies, but they were not the main regulation of GPX4.

## 5. Conclusions

In conclusion, HR is a pathological condition that induces oxidative stress, lipid peroxidation, and ferroptosis to cardiomyocytes. Carvedilol under HR upregulates GPX4, *FTH1*, and *FTL1* expression to resist lipid peroxidation and ferroptosis accompanied with the metabolic upregulation of cysteine and methionine metabolism and GSH metabolism in cardiomyocytes. These findings provide a potential therapeutic approach for Carvedilol utilization in clinical applications and further development in the area of heart diseases.

## Figures and Tables

**Table 1:** List of genes and corresponding forward and reverse primer sequences.

Gene	Forward (5′-3′)	Reverse (5′-3′)
mouse *GSS*(NM_008180)	5′-CAAAGCAGGCCATAGACAGGG-3′	5′-AAAAGCGTGAATGGGGCATAC-3′
mouse *GSR*(NM_010344)	5′-GACACCTCTTCCTTCGACTACC-3′	5′-CCCAGCTTGTGACTCTCCAC-3′
mouse *G6PD*(NM_008062)	5′-CACAGTGGACGACATCCGAAA-3′	5′-AGCTACATAGGAATTACGGGCAA-3′
mouse *NRF2*(BC026943)	5′-CTGAACTCCTGGACGGGACTA-3′	5′-CGGTGGGTCTCCGTAAATGG-3′
mouse *HO-1*(NM_010442)	5′-GCCGAGAATGCTGAGTTCATG-3′	5′-TGGTACAAGGAAGCCATCACC-3′
mouse *FTH1*(NM_010239)	5′-CAAGTGCGCCAGAACTACCA-3′	5′-GCCACATCATCTCGGTCAAAA-3′
mouse *FTL1*(NM_010240)	5′-CCATCTGACCAACCTCCGC-3′	5′-CGCTCAAAGAGATACTCGCC-3′
mouse *beta-Actin*(NM_007393)	5′-GGCTGTATTCCCCTCCATCG-3′	5′-CCAGTTGGTAACAATGCCATG-3′

## Data Availability

The raw data supporting the conclusions of this article will be made available by the authors upon request.
